# Superior Mesenteric Vein Thrombosis Secondary to Oral Contraceptive Use

**DOI:** 10.1155/2012/969130

**Published:** 2012-09-29

**Authors:** Heather Stewart, Michael T. Flannery, Deborah A. Humphrey

**Affiliations:** ^1^Division of Pediatric Nephrology, University of North Carolina Chapel Hill, Chapel Hill, NC 27514, USA; ^2^Department of Internal Medicine, College of Medicine, University of South Florida, Morsani, Tampa, FL 33612, USA

## Abstract

Superior mesenteric vein thrombosis (SMVT) is a rare yet frequently fatal cause of intestinal ischemia. Despite its severe consequences, SMVT often presents with nonspecific symptoms such as nausea, vomiting, and abdominal pain. It can occur with or without gastrointestinal bleeding, and symptoms may be present for hours to weeks. Physical exam can vary from a benign to an acute abdomen. The are no specific diagnostic laboratory studies for the presence of MVT, and it can be an incidental finding of computed tomography or ultrasound. Patients at risk for MVT include those with a history of a hypercoagulable state or secondary cases such as sepsis, gastrointestinal malignancy, liver disease, pancreatic pathology, abdominal surgery and medications. The authors present a case of a patient presenting with acute abdominal pain and ultimately a SMVT secondary to oral contraceptives by exclusion.

## 1. Case Report

A 31-year-old white female presented with a six-day history of abdominal pain and bloating. She described the pain as dull and crampy, with occasional nausea; however, there was no vomiting, diarrhea, fever, chills, or weight loss. At the onset of her symptoms her primary care physician prescribed Lansoprazole 15 mg daily and Simethicone 125 after meals which offered no relief of her symptoms.

The patient was a G1P1001 and experienced no complications with her first pregnancy. There was no history of miscarriages. She denies any history of personal thrombosis, travel, or recent surgery. Her only other medication was an oral contraceptive; ethinyl estradiol 0.02 mg and levonorgestrel 100 ug for which she had been taking for 7 years. She denies any use of tobacco, alcohol, or illicit drug use. There was a remote history of a deep venous thrombosis in her maternal grandfather, following a surgical procedure.

The physical exam on admission demonstrated that she was afebrile with normal vital signs. The remainder of the exam was normal except for deep epigastric tenderness on palpation without signs of peritonitis or distention. The laboratory evaluation revealed a leukocyte count of 9.9 × 10^9^/L, hemoglobin of 11.3 g/L and a platelet count of 320 × 10^9^/L. The remainder of her studies including a complete metabolic profile and a urinalysis were normal. Her erythrocyte sedimentation rate was 44 mm/h and her urine beta HCG was negative.

**Figure 1:**
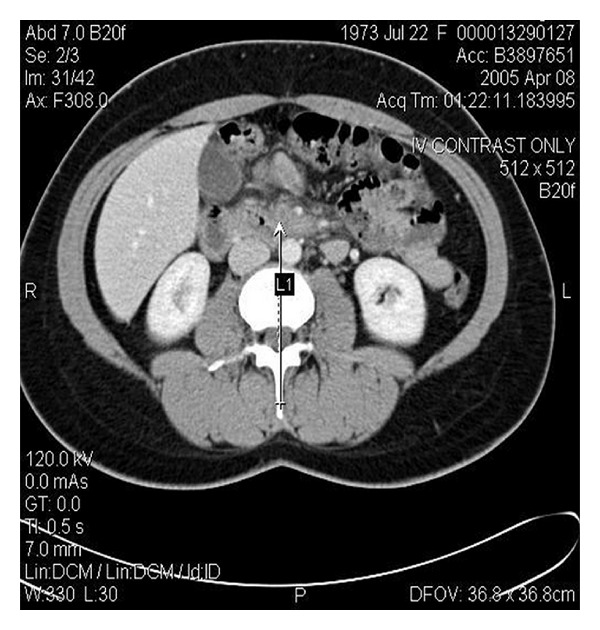
Contrasted abdominal computed tomography demonstrating partial thrombosis of the superior mesenteric vein as noted by the arrow.

A computed tomography of the abdomen revealed evidence of superior mesenteric venous (SMV) thrombosis (see arrow Figure 1). Extremity dopplers showed no evidence of deep venous thrombosis. Subsequently, a magnetic resonance arterial study with venous phase imaging was performed which demonstrated a normal aorta, celiac axis, and superior mesenteric artery, The venous phase showed that the splenic and portal veins appeared normal; however, there was a partial occlusion of the SMV. The patient was started on low molecular weight heparin and warfarin and the following studies were obtained: normal protime and partial thromboplastin time, normal homocysteine level, negative studies for factor 2 and 5 mutations, negative lupus anticoagulant and cardiolipin studies, normal antithrombin III and Protein C and S activity, a negative antinuclear antibody screen and human immunodeficiency viral studies. The patient had a negative sucrose screen for paroxysmal nocturnal hemoglobinuria.

## 2. Discussion

We report a case of contraceptive induced MVT. The earliest description of MVT was in 1895, and the first case of MVT related to oral contraceptives (OCs) was reported in 1963 [1, 2]. MVT related to OC use accounts for 4-5% of all MVT's [3]. The adverse effects of estrogen-progestin OC are thought related to the estrogen component. Major complications include thromboembolic disease, venous hypertension, liver tumor, especially hepatocellular adenomas and rarely colitis [3]. OC-induced thrombosis can be venous or arterial in the systemic, pulmonary, and splanchnic circulations. Progestins are associated with arterial occlusion, in contrast, estrogens can produce both venous and arterial complications [3].

Thrombogenicity correlates with the dose of the estrogen in the OC [3]. A patient's risk of developing OC-induced thrombosis does not increase with the duration of OC use; however, it does increase with age or tobacco use [3]. Risks of a thromboembolic complication decrease within 1 month of discontinuing the OC, therefore, it is recommended that OCs be discontinued at least 1 month prior to major elective surgery [3].

Other factors such as connective tissue disease, hypertension, and tobacco use increase a patient's risk of developing a thrombosis. Women with hereditary antithrombin III deficiency are at increased risk of developing a thrombosis when taking OCs. Therefore, OC use in such patients is contraindicated with this disorder [4]. Data regarding the use of OC in women with protein C deficiency as an increased risk of thrombosis is unclear [5, 6]. Lupus anticoagulant antibodies have not been associated with an increased risk of thrombosis in women taking OCs [7]. The presence of factor 5 Leiden mutations may increase the risk of thrombosis [7].

The mechanisms accounting for the development of thromboembloic phenomena in OC users remain unclear. Several mechanisms have been postulated; (1) OCs may induce a hypercoagulable state by acceleration of the internal and external pathways of the coagulation cascade or by reduction of Antithrombin IIII levels. However, none of these are predictive in discriminating which women will develop a thrombosis, (2) OCs may induce antifibrinolytic activity by decreasing spontaneous fibrinolysis, (3) OCs may induce intimal hyperplasia [4].

Few case reports have documented SMVT without some infarction [4]. Postmortem series suggest that approximately 50% of patients with SMVT do not develop bowel infarction [4]. In one case, the patient was found to have mesenteric nodules and perivascular fibrosis with evidence of transient subacute ischemia without bower infarction [4].

In our case, the patient's abdominal pain was related to SMVT as a result of OC use by exclusion of other normal studies. SMVT is an uncommon disease that carries a significant morbidity and mortality rate that must be considered in the differential diagnosis of abdominal pain in child bearing women on OC, especially those who have been using OCs for more than one year and have personal history of deep venous thrombosis without traditional risk factors.
